# Supplementary material for: Factors associated with accelerometer measured movement behaviours among White British and South Asian children aged 6–8 years during school terms and school holidays
Source: BMJ Open. 2019 Aug 18;9(8):e025071. doi: 10.1136/bmjopen-2018-025071 (PMC6701686; doi:10.1136/bmjopen-2018-025071)
Supplement: Author's manuscript [file bmjopen-2018-025071.draft_revisions.pdf]

BMJ Open is committed to open peer review. As part of this commitment we make the peer review history of every article we publish publicly available.

When an article is published we post the peer reviewers' comments and the authors' responses online. We also post the versions of the paper that were used during peer review. These are the versions that the peer review comments apply to.

The versions of the paper that follow are the versions that were submitted during the peer review process. They are not the versions of record or the final published versions. They should not be cited or distributed as the published version of this manuscript.

BMJ Open is an open access journal and the full, final, typeset and author-corrected version of record of the manuscript is available on our site with no access controls, subscription charges or pay-per-view fees (http://bmjopen.bmj.com).

If you have any questions on BMJ Open's open peer review process please email info.bmjopen@bmj.com

BMJ Open

Factors associated with accelerometer measured movement behaviours among 6-to-8-year-old White British and South Asian children during school terms and school holidays

Journal:	*BMJ Open*
Manuscript ID	bmjopen-2018-025071
Article Type:	Research
Date Submitted by the Author:	02-Jul-2018
Complete List of Authors:	Nagy, Liana; Oxford Brookes University Faculty of Health and Life Sciences; University of Bradford<br>Faisal, Muhammad; University of Bradford,<br>Horne, Maria; University of Leeds<br>Collings, Paul; Bradford Teaching Hospitals NHS Foundation Trust,<br>Barber, Sally; Bradford Institute for Health Research<br>Mohammed, Mohammed ; University of Bradford, Faculty of Health Studies
Keywords:	movement behaviour, children, school holidays, accelerometry, primary school, ethnicity

SCHOLARONE™
Manuscripts

For peer review only

Factors associated with accelerometer measured movement behaviours among 6-to-8-year-old White British and South Asian children during school terms and school holidays

Liana C Nagy[1,2,3], Muhammad Faisal[1,5], Maria Horne[2,4], Paul J Collings[2], Sally E Barber[1,2] and Mohammed A Mohammed[1,2,5]

[1] University of Bradford, Faculty of Health Studies, Richmond Road, Bradford, BD7 1DP

[2] Bradford Institute for Health Research, Bradford Teaching Hospitals Foundation Trust, Bradford, BD9 6RJ

[3] Oxford Brookes University, Faculty of Health and Life Sciences, Jack Straws Lane, Oxford, OX3 0PL

[4] University of Leeds, School of Healthcare, Leeds, LS2 9JT

[5] Yorkshire & Humberside Academic Health Sciences Network

Corresponding author: Liana Nagy

email: lnagy@brookes.ac.uk

Telephone: 01865485282

List of co-authors:

Muhammad Faisal email: M.Faisal1@bradford.ac.uk

Maria Horne email: M.Horne@leeds.ac.uk

Paul James Collings email: paul.collings@bthft.nhs.uk

Sally Elizabeth Barber email: sally.barber@bthft.nhs.uk

Mohammed Amin Mohammed email: M.A.Mohammed5@bradford.ac.uk

Word count: 5260

For peer review only

Abstract

Objectives: To investigate factors associated with movement behaviours among 6-to-8-year old White British (WB) and South Asian (SA) children during school terms and holidays.

Design: Cross-sectional.

Setting: Three primary schools from the Bradford area.

Participants: One-hundred-and-sixty WB and SA children aged 6-to-8-years, wore triaxial accelerometers during all waking hours and overnight for at least 7 days during different data collection periods across three seasons between June 2016-to-May 2017.

Primary and secondary outcomes: Sedentary behaviour (SB), light physical activity (LPA) and moderate-to-vigorous physical activity (MVPA).

Data were analysed using multivariate mixed effects multilevel modelling with robust standard errors.

Factors of interest were: ethnicity, holiday/term, sex, socio-economic status (SES), weight status, weekend/weekday and season.

Results: One-hundred-and-eight children provided 1167 valid days of data. Fifty nine percent of children were WB(n=64) and 41%(n=44) were SA. Boys spent more time in MVPA (11 min/d, p=0.017) compared to girls and SA children spent more time in SB (40 minutes, p=0.017) compared to WB children in adjusted models. Less time (9minutes, p=0.012) was spent in LPA during school terms compared to school holidays. Higher SES children were more sedentary (45 minutes, p=0.036) and less active than low SES children (p=0.005). Children were less active (-17 minutes MVPA, p<0.001; -35 LPA, p<0.001) and more sedentary (56 minutes SB) during winter compared to spring. Children spent more time in MVPA (5 minutes, p=0.036) during weekend compared to weekdays. Overweight children spent less time in SB (-28 minutes, p=0.022) and more time in LPA (24 minutes, p=0.023) than normal weight children

Conclusion: The results of our study suggest that significant child level factors associated with movement behaviours are ethnicity, sex, weight status and SES. Significant environmental factors are weekends, school holidays and seasonality. Interventions to support health enhancing movement behaviours may need to be tailored around these factors.

For peer review only - http://bmjopen.bmj.com/site/about/guidelines.xhtml

Article summary

Strengths and limitations (five bullet points relating to the methods)

- First study in Europe to evaluate school terms and school holidays objectively-measured physical activity data
- Includes White British and South Asian children
- Participants selected from high and low socioeconomic areas
- The use of accelerometers underestimate water based activities
- The demographics of the population might limit the generalisability of the results

For peer review only

Introduction

High levels of sedentary behaviour (SB) and low levels of physical activity (PA) are common among primary school children and have become a public health concern due the negative impact of excessive SB and insufficient PA on physical and mental health (1–3).

More recently, researchers in the field of movement behaviour have started to investigate different compositions of behaviours to identify those compositions which are most health enhancing (4–7). For example, Saunders et al. (8), investigated a reduction in light physical activity (LPA) or SB versus an increase in moderate to vigorous physical activity (MVPA) and concluded that children characterized by low SB, high PA and high levels of sleep have more desirable health outcomes(8). Public health messages are consistent with these ideas and have started adopting slogans such as "Move more, sit less" to promote positive health behaviours in adults and children (6). This indicates that movement behaviour should be considered as a whole instead of focusing on PA or SB separately.

Within the UK, the majority of studies in the field of child movement behaviour have been conducted with White British (WB) children (9–13) and very few have considered ethnic differences between WB and South Asian (SA) children (14–16). The SA population accounts for nearly 8% of the UK population and the percentage rises up to 44% in various cities in the UK. Most of the existing research has reported higher levels of SB and/or lower levels of MVPA for SA children (15,17) although some discrepancies exist. A systematic review (18) on the correlates and determinants of sedentariness included 60 studies between 1999-2006 and concluded, there were no ethnic differences, albeit on the basis of inconclusive results.

Since the SA population is at high risk of developing diabetes and cardiovascular ill health (19–21), including SA participants in studies merits further investigation as the findings may inform the design and development of interventions.

For peer review only - http://bmjopen.bmj.com/site/about/guidelines.xhtml

Although children spend about 20% of the calendar year in school holidays few studies have considered movement behaviours during the school holidays. It has been hypothesized that PA would decrease while SB would increase during the school holidays, when compared to the school terms (22,23) because the unstructured time in holidays is more likely to foster an increase in SB and decrease in PA (24).

The aim of this study was to investigate factors associated with accelerometer measured movement behaviour among WB and SA primary school aged children in Bradford area, during the school terms and school holidays.

Methods

Ethical approval for the study was obtained from the University of Bradford Ethical Committee.

Four- hundred and ninety-two, 6- to- 8-year-old children with a mean age of 7.52 (SD=0.5) from three schools with a Bradford postcode were invited to participate in this cross-sectional study between July 2016 and May 2017. One- hundred and sixty-two were consented by parents. The schools were either predominantly WB and or predominantly SA and included children from various socioeconomic areas, with Index of Multiple Deprivation (IMD) between 1 to 8. Bradford is the 6th largest city in the UK and the third with the highest population of under 16 -year- old (25). It is a highly deprived city and the health outcomes of the population are considerably worse compared with the rest of the UK (26,27)

Data were collected at five time points: summer (term; n=55 consented /152 invited), winter (term and holiday; n=69/154) and spring (term and holiday; n=38/186). Primary school aged children were invited to wear an Actigraph GT3X+ triaxial accelerometer (Actigraph, Pensacola, FL, USA). The Actigraph was attached on an elastic band around their waist on the right hip. Participating children were asked to wear the accelerometer during all waking hours and overnight for at least 7 days. Children who participated in the winter and spring data collections were invited to wear the device during school term and school holiday with 1-2-week break between the two-data collections. Therefore, the study comprised of three different samples of children according to the season of data collection: summer, winter and spring.

Procedure and measurements

Research measurements taken by trained staff included height (cm) (wall mounted stadiometer: Seca UK, Birmingham UK), weight (Kg), body composition (measured with Tanita scales TBF-300 MA, Tokyo, Japan and Tanita scales BC-418 MA, Tokyo, Japan), and waist circumference (Seca anthropomentry tape) measured just above the exposed naval. Height was recorded to the nearest 0.1 cm and 0.1 kg, with shoes and socks removed. BMI values were calculated (weight (kg)/height (m)^2) and BMI (body mass index) z-

For peer review only - http://bmjopen.bmj.com/site/about/guidelines.xhtml

scores were calculated for each participant using the British growth reference(28). Weight categories were derived from BMI percentiles using Freeman et al. (29) recommendations.

Parents completed a demographics which included child postcode, date of birth, ethnicity and parent's education. Based on each child home postcode, the Index of Multiple Deprivation (IMD) was determined and collapsed in three levels of SES: low (IMD1-2), medium (IMD3-5) and high (IMD>6)(30).

Data management and processing

The accelerometer was set to record data at a sampling rate of 60Hertz. Raw accelerometer data were downloaded and collapsed into 15s epoch files. The Evenson (31) cut-points were selected for this study because they have been reported to be among of the most accurate to estimate MVPA for children aged 6-to-8-year-old (32). The calibration studies for these cut off points meet the 4 item criteria by Freedson et al. (33) and Welk et al (34). The choice for 15s epoch length was in line with the calibration studies for this population group age (31). Non-wear time was defined as ≥ 20 minutes of consecutive zero counts as they are the most reported ; 10-30 minutes is one of the most reported non-wear periods (35).

The minimum valid wear time for inclusion in the analysis was at least 10 hours on at least three-week days and at least one weekend day (Saturday or Sunday) for any data collection. This criteria of valid data inclusion is reported to have high reliability for estimates of PA (36). Sleep time was removed from the data each day between 11 pm and 5 am similarly to other studies for primary school aged children (37,38) but also to take into consideration that the majority of SA children in our study were Muslims who are encouraged to wake up for the dawn prayer, which during spring and summer is around 5 am(39).

Data Analysis

Statistical analyses were conducted using Stata v.13.1 (40). The three outcome variables were SB, LPA and MVPA. Data were analysed using multivariate linear mixed effects multilevel modelling with robust standard errors to mitigate the impact of heteroskedasticity from skewed outcome variables. All models adjusted for the following factors: wear time, ethnicity, holiday/term sex, SES, weight status, weekend/weekday, season and school. P-values of < 0.05 were considered to be statistically significant. Data are displayed as mean (± SD) and mean (95% Confidence Intervals (CI) in the text and tables.

For peer review only - http://bmjopen.bmj.com/site/about/guidelines.xhtml

Results

Out of 492 invited children, consent/assent was available for 160 (32.5%). A further 54 children were excluded because they did not meet the inclusion criteria for valid data leaving 108 (67.5%, 108/160) children who had valid data included in the analyses.

Table 1 shows the profile of the children. The mean age of the 108 children is 7.52 old and the male/female ratio was 0.96 (53/55). Out of the 108 children, 59%(64/108) were WB and 41% (44/108) were SA. Forty-one (38%; 41/108) children were from the 20% most deprived areas (IMD 1-2), however that was the case for the majority of SA (80%; 35/44) children. About three-quarters of children (77%, 83/108) were of normal weight and 23% (25/108) were overweight and obese although a higher percentage (27%; 12/44) of SA children were overweight and obese (Table 1).

Table 1 : Characteristics of children whose data was included in the analyses.

Characteristic	White British N=64 (59%)	South Asian N=44 (41%)	All children N=108
Age, mean (sd)	7.52(0.50)	7.61 (0.49)	7.52 (0.50)
Male (%)<br>Female (%)	32 (50)<br>32 (50)	21 (48)<br>23 (52)	53 (49)<br>55(51)
Low SES (%)<br>Medium SES (%)<br>High SES (%)	6 (9.38)<br>40(62.50)<br>18 (28.13)	35 (79.55)<br>9 (20.35)<br>0 (0.0)	41 (38)<br>49 (45)<br>18 (17)
Zbmiuk, mean (sd)	0.31 (.98)	0.38 (1.34)	0.34(1.14)
Normal weight (%)<br>Overweight (%)<br>Obese (%)	51 (80)<br>9(14)<br>4(6)	32 (73)<br>3 (7)<br>9 (20)	83 (77)<br>12 (11)<br>13 (12)

The 108 children who had valid data provided a total of 1167 terms and holidays days. Children (SA and WB combined) had on average 901 minutes of daily valid wear of which 539 minutes (60%) were spent SB, 296 minutes (33%) in LPA and 67 minutes (7%) were MVPA.

Table 2 gives an overview of the unadjusted averages stratified by ethnicity, school terms and school holidays. The unadjusted results in the whole data set identified slightly higher proportions of SB (61%; 550 minutes) and LPA (33%; 292 minutes) but lower proportions of MVPA (6%; 57 minutes) for SA children

For peer review only - http://bmjopen.bmj.com/site/about/guidelines.xhtml

compared to WB children. A similar pattern was observed when stratifying the results by term and holiday (Table 2).

For peer review only - http://bmjopen.bmj.com/site/about/guidelines.xhtml

Table 2: Unadjusted data sets averages by ethnicity.

	Whole data				School terms				School holidays			
Whole data set	WB N=64 Mean (sd)	% of wear time	SA N=34 Mean (sd)	% of wear time	WB N=56 Mean (sd)	% of wear time	SA N=39 Mean (sd)	% of wear time	WB N=40 Mean (sd)	% of wear time	SA N=23 Mean (sd)	%of wear time
Total number of valid days	684	n/a	473	-	369	n/a	269	-	315	n/a	214	-
Valid wear time in minutes	888.78 (117.16)	n/a	899.85 (110.55)	-	876.66 (105.60)	n/a	902.12 (120.55)	-	885.88 (105.83)	n/a	915.03 (85.22)	-
Total SB in minutes	532.05 (109.13)	60	550.13 (117.57)	61	532.42 (104.44)	61	553.05 (113.56)	61	522.10 (11.2)	60	564.55 (76.89)	61
Total LPA	288.73 (66.64)	32	292.27 (52.02)	33	275.54 (64.78)	31	289.27 (55.03)	32	299.90 (67.14)	33	296.90 (56.47)	32
Total MVPA	67.99 (33)	8	57.44 (37.97)	6	68.95 (31.49)	8	59.80 (34.08)	7	63.85 (31.44)	7	53.57 (24.88)	7

For peer review only - http://bmjopen.bmj.com/site/about/guidelines.xhtml

Table 3 reports findings from statistical models for each outcome variable: SB, LPA and MVPA following mutual adjustment for predictors of interest. Once wear time, term, ethnicity, SES, school, season, sex, weekend and weight status were adjusted for, significant differences were identified for SB in relation to 4 factors: ethnicity, SES, seasonality and overweight; for LPA in relation to 4 factors: term, SES, seasonality and overweight; and for MVPA in relation to 3 factors: sex, seasonality and weekend (Table 3).

Specifically, boys spent significantly more time in MVPA (11 min/d) compared to girls and SA children spent more time in SB (40 minutes) compared to WB children (p=0.017). More time (8 minutes SB) was spent in SB and less time in LPA (9 minutes) during school terms compared to school holidays but only LPA was statistically significant (p=0.012). High SES children were more sedentary (45 minutes SB) than low SES children (p=0.005). Children were less active (-17 minutes MVPA, -39 LPA) and more sedentary (55 minutes SB) during winter compared to spring (p=0.036; p<0.001; p<0.001). Children spent more time in MVPA (5 minutes) during weekend compared to weekdays (p=0.003). Overweight children spent more time in LPA (24 minutes) and less (-28 minutes) in SB than normal weight children (p=0.023; p=0.022).

Nearly half the children (46%, 49/108) did not meet the recommended guidelines of 60 minutes of MVPA per day in any of the days included in the analyses: 50% (22/44) of SA and 57 % (27/64) of WB children.

For peer review only

For peer review only - http://bmjopen.bmj.com/site/about/guidelines.xhtml

Table 3: Model coefficients for three movement behaviour outcomes based on the whole data set (terms and holidays days) (CI 95%).

Covariates	SB Mean (min to max) p-value	LPA Mean (min to max) p-value	MVPA Mean (min to max) p-value
South Asian (reference group: White British)	39.52 (6.95 to 72.09) p = 0.017	-23.98 (-48.18 to 0.23) p = 0.052	-15.62 (-35.04 to 3.79) p = 0.115
School Term	8.03 (-1.41 to 17.47) p = 0.095	-8.64 (-15.38 to -1.89) p = 0.012	0.59 (-3.85 to 5.02) p = 0.795
Male	-0.73 (-18.45 to 16.99) p = 0.936	-10.01 (-23.2 to 3.18) p = 0.137	10.73 (1.93 to 19.53) p = 0.017
Medium SES	15.86 (-6.1 to 37.83) p = 0.157	-17.3 (-31.68 to -2.92) p = 0.018	1.62 (-12.06 to 15.31) p = 0.816
High SES	44.56 (13.75 to 75.38) p = 0.005	-31.69 (-56.03 to -7.34) p = 0.011	-12.72 (-28.21 to 2.76) p = 0.107
Summer	13.85 (-15.23 to 42.93) p = 0.351	-11.98 (-33.44 to 9.48) p = 0.274	-1.9 (-18.75 to 14.94) p = 0.825
Winter	55.33 (28.02 to 82.64) p < 0.001	-38.66 (-59.35 to -17.97) p < 0.001	-16.73 (-32.35 to -1.12) p = 0.036
Weekend	-6.02 (-15.44 to 3.41) p = 0.211	1.39 (-5.66 to 8.43) p = 0.7	4.66 (0.31 to 9.01) p = 0.036
Overweight	-27.61 (-51.29 to -3.92) p = 0.022	23.82 (3.34 to 44.29) p = 0.023	3.76 (-7.12 to 14.64) p = 0.499
Obese	-11.89 (-45.98 to 22.2) p = 0.494	18.81 (-6.97 to 44.58) p = 0.153	-6.88 (-21.05 to 7.29) p = 0.341

* The shaded cells in the table identify statistically significant difference.

Discussion

Summary of findings

Overall, children spent a large proportion of time in SB, approximately 60-62% (550 to 564 min/d), followed by LPA, approximately 31-33% (276 to 300 minutes) and a smaller proportion in MVPA, 6-8 % (57 to 69 minutes. We also identified other key findings: 1) SA children were more sedentary that WB children; (2)

For peer review only - http://bmjopen.bmj.com/site/about/guidelines.xhtml

There were no apparent differences for SB and MVPA, yet LPA was lower during term compared to school holiday; 3) Children had more MVPA during weekend compared to weekdays; 4) Children were markedly more sedentary and less active during the winter compared to spring and summer; and 5) children from high SES were more sedentary and less active (LPA) compared to children from low SES.

Comparison to other studies

We found that SA children spent 40 more min/d in SB compared to WB children (p=0.031)which is similar to the results of other studies evaluating ethnic differences in PA and SB in WB and SA , 8 to 9 year old children (41) and 9 to 11 year old girls in England(42). Both studies identified that SA children were less active and more sedentary compared to WB children. The results of the Millennium study also identified that SA children were the most sedentary and least active when compared to WB or children of African origin (43).

There were significant differences between the school term and the school holidays, with 8.64 fewer min/d of LPA spent during the school terms time compared to the school holidays (p= 0.012). Our finding of higher SB (not statistically significant) and lower LPA during the school term is contrary to previous reports of higher SB and lower PA during school holidays (44). Currently the majority of evidence on interventions aimed at increasing PA and/or reducing SB focuses on school term time only (45–47). Our results imply that interventions to promote positive movement behaviour are needed also during holidays as high levels of SB have been recorder during both occasions. From a practical point of view of data collection and interventions, children are more accessible en masse during the school term, however the school holiday period should be considered as appears to be a critical time period for some children.

The fact that seasonality was identified as a consistent significant factor in all movement behaviour outcomes (SB higher and LPA, MVPA lower during the winter season compared to spring and summer) is similar to the study of Atkin et al (48) which identified elevated levels of SB in winter and autumn for children aged 7-years and suggested the need for interventions during autumn and winter. Similarly, a study involving over 1000 primary school aged Danish children, (49) found higher SB and lower MVPA during winter compared to spring. Unlike our study, Hjort et al. (49) identified higher SB and lower MVPA at the weekend, which is the case in most studies (10,16,50) with primary school children.

Overall, children spent a large proportion of time in SB, followed by LPA, and a smaller proportion in MVPA, 6-8 % (57 to 69 minutes) (Table 2). Although the children in our study had high levels of SB, the average level of MVPA was higher than compared with other studies (43,49). Results from the Millennium Study(43) on 13000, 7 year of children identified an average of 38 minutes/ day spent in MVPA. The Millennium study

For peer review only - http://bmjopen.bmj.com/site/about/guidelines.xhtml

however had a different definition of a valid day and included days with >6 hours or valid wear time. It is possible that the higher levels of MVPA in our study are related data being collected over three weeks of summer and spring and only two weeks of winter.

Strengths and limitations

The strength our study is related to uniqueness of the sample which included SA and WB children from varied SES areas, and we also included data during the school holidays. To our knowledge this is the first study in the UK to take into consideration school holidays.

The sample of the study was modest which means that we may have been underpowered to detect smaller effect sizes. However, our sample size was similar to a Japanese study conducted by Tanaka and colleagues which considered the summer holidays accelerometry and included 98 children in the data analysis(22). A larger study which considered PA and SB during school holidays was conducted in the USA and included 406 children but the data were collected over 20 months and PA and SB were self-reported(23).

Despite aiming to include children from various SES, this was challenging because in Bradford the majority of SA children were from the poorest areas with only 20% of SA children from medium SES and 0% from high SES.

Another limitation of the study could be the definition of sleep and the 5 am awake time. It is possible that some sleeping hours may have been misclassified as SB, thereby overestimating SB. Our choice for the 5am awake time was made in the context of SA children, majority of Muslim faith, who are encouraged to wake up around this time for the morning prayer. Future studies should consider designs that allow seasonal comparisons within children measured throughout the year; the best we could do is compare seasonal PA between different groups of children.

Conclusion

The results of our study suggest that children spent high proportions of their time in SB. Significant child level factors associated with movement behaviour were ethnicity, sex, weight status and SES. Significant environmental factors were, weekend holidays and seasonality. SA children were more sedentary and less active than WB children. Interventions to reduce SB and increase PA are needed over school term and school holidays. Interventions aimed at reducing SB and increasing PA are needed across all seasons but especially during the winter when SB is higher and PA is lower.

For peer review only - http://bmjopen.bmj.com/site/about/guidelines.xhtml

References:

1. LeBlanc AG, Spence JC, Carson V, Connor Gorber S, Dillman C, Janssen I, et al. Systematic review of sedentary behaviour and health indicators in the early years (aged 0–4 years). Appl Physiol Nutr Metab. 2012;37(4):753–72.
2. Tremblay M, LeBlanc A, Kho M, Saunders T, Larouche R, Colley R, et al. Systematic review of sedentary behaviour and health indicators in school-aged children and youth. Int J Behav Nutr Phys Act. 2011;8(1):98–119.
3. Hancox RJ, Milne BJ, Poulton R. Association between child and adolescent television viewing and health: A longitudinal birth cohort study. Lancet. 2004;364(9430):257–62.
4. Ekelund U, Luan J, Sherar LB, Esliger DW, Griew P, Cooper A. Moderate to vigorous physical activity and sedentary time and cardiometabolic risk factors in children and adolescents. JAMA. 2012;307.
5. Tremblay MS, Carson V, Chaput J-P, Connor Gorber S, Dinh T, Duggan M, et al. Canadian 24-Hour Movement Guidelines for Children and Youth: An Integration of Physical Activity, Sedentary Behaviour, and Sleep. Appl Physiol Nutr Metab. 2016;41(6 (Suppl. 3)).
6. Dumuid D, Pedišić Ž, Stanford TE, Martín-Fernández J-A, Hron K, Maher CA, et al. The compositional isotemporal substitution model: A method for estimating changes in a health outcome for reallocation of time between sleep, physical activity and sedentary behaviour. Stat Methods Med Res [Internet]. SAGE Publications Ltd STM; 2017 Nov 20;0962280217737805.
7. Pedišić Ž, Dumuid D, Olds TS. Integrating sleep, sedentary behaviour, and physical activity research in the emerging field of time-use epidemiology: Definitions, concepts, statistical methods, theoretical framework, and future directions. Kinesiology. 2017;49(2):135–45.
8. John Saunders T, Ellen Gray C, Joan Poitras V, Chaput J-P, Janssen I, Katzmarzyk PT, et al. Combinations of physical activity, sedentary behaviour and sleep: relationships with health indicators in school-aged children and youth 1. Appl Physiol Nutr Metab Appl Physiol Nutr Metab. 2016;41(June):283–93.
9. King AC, Parkinson KN, Adamson AJ, Murray L, Besson H, Reilly JJ, et al. Correlates of objectively measured physical activity and sedentary behaviour in English children. Eur J Public Health. 2011;21(4):424–31.
10. Collings PJ, Wijndaele K, Corder K, Westgate K, Ridgway CL, Dunn V, et al. Levels and patterns of objectively-measured physical activity volume and intensity distribution in UK adolescents: the ROOTS study. Int J Behav Nutr Phys Act [Internet]. 2014;11(1):23.

For peer review only - http://bmjopen.bmj.com/site/about/guidelines.xhtml

11. Chinapaw MJM, Proper KI, Brug J, van Mechelen W, Singh AS. Relationship between young peoples' sedentary behaviour and biomedical health indicators: A systematic review of prospective studies. Obes Rev. 2011;12(7).

12. Nelson MC, Neumark-Stzainer D, Hannan PJ, Sirard JR, Story M. Longitudinal and secular trends in physical activity and sedentary behavior during adolescence. Pediatrics 2006;118.

13. Townsend N, Wickramasinghe K, Williams J, Bhatnagar P, Rayner M. Physical activity statistics. British Heart Foundation. 2015. 1-128 p.

14. Hornby-Turner YC, Hampshire KR, Pollard TM. A comparison of physical activity and sedentary behaviour in 9-11 year old British Pakistani and White British girls: A mixed methods study. Int J Behav Nutr Phys Act. 2014;11(1):74.

15. Barber SE, Kelly B, Collings PJ, Nagy L, Bywater T, Wright J. Prevalence , trajectories , and determinants of television viewing time in an ethnically diverse sample of young children from the UK. Int J Behav Nutr Phys Act. 2017;14:88.

16. Eyre ELJ, Duncan MJ, Smith EC, Matyka KA. Objectively measured patterns of physical activity in primary school children in Coventry: the influence of ethnicity. Diabet Med. England; 2013 Aug;30(8):939–45.

17. Pollard B, Johnston M, Dieppe P. What do osteoarthritis heath outcome instruments measure? Impairment, activity limitation or participation restriction? J Rheumatol. 2006;33.

18. Horst KVANDER, Twisk JOSWR, Mechelen WVAN. A Brief Review on Correlates of Physical Activity and Sedentariness in Youth. 2005;(January):1241–50.

19. H.M. M, N. C, J.H. F. Mortality and morbidity from diabetes in South Asians and Europeans: 11-year follow-up of the Southall Diabetes Survey, London, UK. Diabet Med 2004 Jul 19;15(1):53–9.

20. Gholap N, Davies M, Patel K, Sattar N, Khunti K. Type 2 diabetes and cardiovascular disease in South Asians. Prim Care Diabetes 2011 Apr 1;5(1):45–56.

21. Bhopal R, Hayes L, White M, Unwin N, Harland J, Ayis S, et al. Ethnic and socio-economic inequalities in coronary heart disease, diabetes and risk factors in Europeans and South Asians. J Public Health. 2002 Jun 1;24(2):95–105.

22. Tanaka C, Reilly JJ, Tanaka M, Tanaka S. Seasonal changes in objectively measured sedentary behavior and physical activity in Japanese primary school children. BMC Public Health. London: BioMed Central; 2016 Sep 13;16(1):969.

23. Staiano AE, Broyles ST, Katzmarzyk PT. School Term vs. School Holiday: Associations with Children's Physical Activity, Screen-Time, Diet and Sleep. Tchounwou PB, editor. Int J Environ Res Public Health [Internet]. MDPI; 2015 Aug 30;12(8):8861–70.

24. Arundell L, Fletcher E, Salmon J, Veitch J, Hinkley T. A systematic review of the prevalence of sedentary behavior during the after-school period among children aged 5-18 years. Int J Behav Nutr Phys Act [Internet]. International Journal of Behavioral Nutrition and Physical Activity; 2016

For peer review only - http://bmjopen.bmj.com/site/about/guidelines.xhtml

Aug;13(1):93.

25. ONS. Census 2011. Census [Internet]. 2011;(May):78. Available from: http://www.ons.gov.uk/ons/guide-method/census/2011/index.html

26. Firth C, Petherick E, Oddie SJ. Infant deaths from congenital anomalies: novel use of Child Death Overview Panel data. Arch Dis Child [Internet]. 2018;archdischild-2017-314256.

27. Wright J, Small N, Raynor P, Tuffnell D, Bhopal R, Cameron N, et al. Cohort profile: The born in bradford multi-ethnic family cohort study. Int J Epidemiol. 2012;42(4):978–91.

28. Cole TJ, Freeman J V, Preece MA. Body mass index reference curves for the UK, 1990. Arch Dis ChildhoodArch Dis Child. 1995;73(73):25–9.

29. Freeman V, Cole TJ, Chinn S, Jones PRM, White EM, Preece MA. Cross sectional stature and weight reference curves for the UK , 1990. 1995;17–24.

30. ONS. The English Indices of Deprivation 2015 Statistical Release. 2015.

31. Evenson KR, Catellier DJ, Gill K, Ondrak KS, McMurray RG. Calibration of two objective measures of physical activity for children. J Sports Sci. 2008;26(14):1557–65.

32. Kim Y, Lee J-M, Peters BP, Gaesser GA, Welk GJ. Examination of different accelerometer cut-points for assessing sedentary behaviors in children. PLoS One [Internet]. Department of Kinesiology, Iowa State University, Ames, Iowa, United States of America. School of Health, Physical Education and Recreation College of Education, University of Nebraska, Omaha, Nebraska, United States of America. School of Nutrition and He; 2014;9(4):e90630–e90630.

33. Freedson P, Pober D, Janz KF. Calibration of accelerometer output for children. In: Medicine and Science in Sports and Exercise. 2005.

34. Welk GJ. Principles of design and analyses for the calibration of accelerometry-based activity monitors. In: Medicine and Science in Sports and Exercise. 2005.

35. Aadland E, Andersen LB, Anderssen SA, Resaland GK. A comparison of 10 accelerometer non-wear time criteria and logbooks in children. BMC Public Health [Internet]. London: BioMed Central; 2018 Mar 6;18:323.

36. Rich C, Geraci M, Griffiths L, Sera F, Dezateux C, Cortina-Borja M. Quality Control Methods in Accelerometer Data Processing: Defining Minimum Wear Time. PLoS One. 2013;8(6):1–8.

37. Sherry A, Pearson N, Ridgers ND, Barber SE, Bingham DD, Nagy LC, et al. Inclinometer measured sitting patterns in 9-10 year old chidlren from a northern UK city. Eur J Public. 2018;in press.

38. Meredith-Jones K, Williams S, Galland B, Kennedy G, Taylor R. 24 h Accelerometry: impact of sleep-screening methods on estimates of sedentary behaviour and physical activity while awake. J Sport Sci. 2015;0414(March).

39. BaHammam AS, Gozal D. Qur'anic insights into sleep. Nat Sci Sleep [Internet]. Dove Medical Press; 2012 Jul 24;4:81–7.

40. StataCorp. Stata Statistical Software: Release 13. 2013. 2013.

For peer review only - http://bmjopen.bmj.com/site/about/guidelines.xhtml

41. E.L.J. E, M.J. D, S.L. B, V.M. C. Low socio-economic environmental determinants of children's physical activity in Coventry, UK: A Qualitative study in parents. Prev Med Reports, Department of Applied Science and Health, Coventry University, Coventry, United Kingdom; 2014;1:32–42.

42. Pollard TM, Hornby-Turner YC, Ghurbhurrun A, Ridgers ND. Differences between 9-11 year old British Pakistani and White British girls in physical activity and behavior during school recess. BMC Public Health [Internet]. BMC Public Health; 2012;12(1):1087.

43. Griffiths LJ, Pouliou T, Rich C, Geraci M, Cortina-Borja M, Sera F, et al. Objectively measured physical activity and sedentary behaviour in UK children of primary school age: a longitudinal cohort study. Lancet [Internet]. Elsevier; 2012 Nov 23;380:S44.

44. Brazendale K, Beets MW, Weaver RG, Pate RR, Turner-mcgrievy GM, Kaczynski AT, et al. Understanding differences between summer vs . school obesogenic behaviors of children : the structured days hypothesis. International Journal of Behavioral Nutrition and Physical Activity; 2017;1–14.

45. Atkin AJ, Gorely T, Biddle SJH, Cavill N, Foster C. Interventions to Promote Physical Activity in Young People Conducted in the Hours Immediately After School : A Systematic Review. 2011;176–87.

46. O'Dwyer M V., Fairclough SJ, Ridgers ND, Knowles ZR, Foweather L, Stratton G. Effect of a school-based active play intervention on sedentary time and physical activity in preschool children. Health Educ Res. 2013;28(6).

47. Sluijs EM, McMinn AM, Griffin SJ. Effectiveness of interventions to promote physical activity in children and adolescents: systematic review of controlled trials. BMJ [Internet]. 2007;335.

48. Atkin AJ, Shard SJ, O HFL, Brage S, Van Sluijs EMF. Seasonal Variation in Children's Physical Activity and Sedentary Time. Med Sci Sports Exerc [Internet]. Lippincott Williams & Wilkins; 2016 Mar 18;48(3):449–56.

49. Hjorth MF, Chaput J-P, Michaelsen K, Astrup A, Tetens I, Sjödin A. Seasonal variation in objectively measured physical activity, sedentary time, cardio-respiratory fitness and sleep duration among 8-11 year-old Danish children: a repeated-measures study. BMC Public Health [Internet]. 2013;13(1):808.

50. Fairclough SJ, Boddy LM, Mackintosh KA, Valencia-Peris A, Ramirez-Rico E. Weekday and weekend sedentary time and physical activity in differentially active children. J Sci Med Sport. 2015;18(4):444–9.

51. Farooq MA, Parkinson KN, Adamson AJ, Pearce MS, Reilly JK, Hughes AR, et al. Timing of the decline in physical activity in childhood and adolescence: Gateshead Millennium Cohort Study. Br J Sports Med [Internet]. 2017 Feb 4;

52. Griffiths LJ, Sera F, Cortina-Borja M, Law C, Ness A, Dezateux C. Objectively measured physical activity and sedentary time: cross-sectional and prospective associations with adiposity in the Millennium Cohort Study. BMJ Open [Internet]. 2016;6(4)

For peer review only

For peer review only - http://bmjopen.bmj.com/site/about/guidelines.xhtml

Funding Statement:

This research was funded by the NIHR Collaboration for Leadership in Applied Health Research and Care Yorkshire and Humber (NIHR CLAHRC YH). www.clahrc-yh.nihr.ac.uk. The views and opinions expressed are those of the authors, and not necessarily those of the NHS, the NIHR or the Department of Health.

Competing interest statement:

None.

Authors contribution:

Study design and research question: LCN, MH, SEB, MAM, PJC

Data collection: LCN, MH, PJC

Data processing and analysis: All authors contributed to discussions on data analysis. Data processing and analysis was carried out by LCN, MAM and MF.

Ongoing discussion and comments: all

Drafted paper and final paper: LN

Patient consent form:

N/A

Data sharing statement:

Data is not available to the public due to participant consent restrictions.

Acknowledgements

We thank all the participants and their families for their time and ongoing participation in the study. We also thank all the researchers involved in data collection.

For peer review only - http://bmjopen.bmj.com/site/about/guidelines.xhtml

STROBE Statement—checklist of items that should be included in reports of observational studies

	Item No	Recommendation	Page No.
**Title and abstract**	1	(*a*) Indicate the study's design with a commonly used term in the title or the abstract	2
		(*b*) Provide in the abstract an informative and balanced summary of what was done and what was found	2
**Introduction**			
Background/rationale	2	Explain the scientific background and rationale for the investigation being reported	3-4
Objectives	3	State specific objectives, including any prespecified hypotheses	4
**Methods**			
Study design	4	Present key elements of study design early in the paper	4-5
Setting	5	Describe the setting, locations, and relevant dates, including periods of recruitment, exposure, follow-up, and data collection	4-5
Participants	6	(*a*) *Cohort study*—Give the eligibility criteria, and the sources and methods of selection of participants. Describe methods of follow-up<br>*Case-control study*—Give the eligibility criteria, and the sources and methods of case ascertainment and control selection. Give the rationale for the choice of cases and controls<br>*Cross-sectional study*—Give the eligibility criteria, and the sources and methods of selection of participants	4
		(*b*) *Cohort study*—For matched studies, give matching criteria and number of exposed and unexposed<br>*Case-control study*—For matched studies, give matching criteria and the number of controls per case	
Variables	7	Clearly define all outcomes, exposures, predictors, potential confounders, and effect modifiers. Give diagnostic criteria, if applicable	5
Data sources/ measurement	8*	For each variable of interest, give sources of data and details of methods of assessment (measurement). Describe comparability of assessment methods if there is more than one group	5
Bias	9	Describe any efforts to address potential sources of bias	5
Study size	10	Explain how the study size was arrived at	6
Quantitative variables	11	Explain how quantitative variables were handled in the analyses. If applicable, describe which groupings were chosen and why	
Statistical methods	12	(*a*) Describe all statistical methods, including those used to control for confounding	5
		(*b*) Describe any methods used to examine subgroups and interactions	
		(*c*) Explain how missing data were addressed	
		(*d*) *Cohort study*—If applicable, explain how loss to follow-up was addressed<br>*Case-control study*—If applicable, explain how matching of cases and controls was addressed<br>*Cross-sectional study*—If applicable, describe analytical methods taking account of sampling strategy	
		(*e*) Describe any sensitivity analyses	

For peer review only - http://bmjopen.bmj.com/site/about/guidelines.xhtml

**Results**			
Participants	13*	(a) Report numbers of individuals at each stage of study—eg numbers potentially eligible, examined for eligibility, confirmed eligible, included in the study, completing follow-up, and analysed	6
		(b) Give reasons for non-participation at each stage	
		(c) Consider use of a flow diagram	
Descriptive data	14*	(a) Give characteristics of study participants (eg demographic, clinical, social) and information on exposures and potential confounders	6
		(b) Indicate number of participants with missing data for each variable of interest	
		(c) *Cohort study*—Summarise follow-up time (eg, average and total amount)	
Outcome data	15*	*Cohort study*—Report numbers of outcome events or summary measures over time	
		Case-control study—Report numbers in each exposure category, or summary measures of exposure	
		Cross-sectional study—Report numbers of outcome events or summary measures	6-11
Main results	16	(*a*) Give unadjusted estimates and, if applicable, confounder-adjusted estimates and their precision (eg, 95% confidence interval). Make clear which confounders were adjusted for and why they were included	6-11
		(*b*) Report category boundaries when continuous variables were categorized	
		(*c*) If relevant, consider translating estimates of relative risk into absolute risk for a meaningful time period	
Other analyses	17	Report other analyses done—eg analyses of subgroups and interactions, and sensitivity analyses	
**Discussion**			
Key results	18	Summarise key results with reference to study objectives	10-11
Limitations	19	Discuss limitations of the study, taking into account sources of potential bias or imprecision. Discuss both direction and magnitude of any potential bias	12
Interpretation	20	Give a cautious overall interpretation of results considering objectives, limitations, multiplicity of analyses, results from similar studies, and other relevant evidence	11-12
Generalisability	21	Discuss the generalisability (external validity) of the study results	11-12
**Other information**			
Funding	22	Give the source of funding and the role of the funders for the present study and, if applicable, for the original study on which the present article is based	17

*Give information separately for cases and controls in case-control studies and, if applicable, for exposed and unexposed groups in cohort and cross-sectional studies.

Note: An Explanation and Elaboration article discusses each checklist item and gives methodological background and published examples of transparent reporting. The STROBE checklist is best used in conjunction with this article (freely available on the Web sites of PLoS Medicine at http://www.plosmedicine.org/, Annals of Internal Medicine at http://www.annals.org/, and Epidemiology at http://www.epidem.com/). Information on the STROBE Initiative is available at www.strobe-statement.org.

For peer review only

For peer review only - http://bmjopen.bmj.com/site/about/guidelines.xhtml

BMJ Open

Factors associated with accelerometer measured movement behaviours among 6-to-8-year-old White British and South Asian children during school terms and school holidays

Journal:	*BMJ Open*
Manuscript ID	bmjopen-2018-025071.R1
Article Type:	Research
Date Submitted by the Author:	21-Mar-2019
Complete List of Authors:	Nagy, Liana; Oxford Brookes University Faculty of Health and Life Sciences; University of Bradford<br>Faisal, Muhammad; University of Bradford,<br>Horne, Maria; University of Leeds Faculty of Medicine and Health, School of Healthcare<br>Collings, Paul; Bradford Institute for Health Research; University of York<br>Barber, Sally; Bradford Institute for Health Research<br>Mohammed, Mohammed ; University of Bradford, Faculty of Health Studies
**Primary Subject Heading**:	Public health
Secondary Subject Heading:	Public health
Keywords:	movement behaviour, children, school holidays, accelerometry, primary school, ethnicity

SCHOLARONE™
Manuscripts

Factors associated with accelerometer measured movement behaviours among 6-to-8-year-old White British and South Asian children during school terms and school holidays

Liana C Nagy[1,2,3], Muhammad Faisal[1,5], Maria Horne[2,4], Paul J Collings[2,6], Sally E Barber[1,2] and Mohammed A Mohammed[1,2,5]

[1] University of Bradford, Faculty of Health Studies, Richmond Road, Bradford, BD7 1DP

[2] Bradford Institute for Health Research, Bradford Teaching Hospitals Foundation Trust, Bradford, BD9 6RJ

[3] Oxford Brookes University, Faculty of Health and Life Sciences, Jack Straws Lane, Oxford, OX3 0PL

[4] University of Leeds, School of Healthcare, Leeds, LS2 9JT

[5]Yorkshire & Humberside Academic Health Sciences Network

[6] University of York, Department of Health Sciences, YO10 5DD

Corresponding author: Liana Nagy

Email: lnagy@brookes.ac.uk

Telephone: 01865485282

List of co-authors:

Muhammad Faisal email: M.Faisal1@bradford.ac.uk

Maria Horne email: M.Horne@leeds.ac.uk

Paul James Collings email: paul.collings@bthft.nhs.uk

Sally Elizabeth Barber email: sally.barber@bthft.nhs.uk

Mohammed Amin Mohammed email: M.A.Mohammed5@bradford.ac.uk

Word count: 3480 excluding tables and references

For peer review only - http://bmjopen.bmj.com/site/about/guidelines.xhtml

Abstract

Objectives: To investigate factors associated with movement behaviours among 6-to-8-year old White British (WB) and South Asian (SA) children during school terms and holidays.

Design: Cross-sectional.

Setting: Three primary schools from the Bradford area, UK.

Participants: One-hundred-and-sixty WB and SA children aged 6-to-8-years old

Primary and secondary outcomes: Sedentary behaviour (SB), light physical activity (LPA) and moderate-to-vigorous physical activity (MVPA) measured by accelerometry during summer, winter and spring and during school terms and school holidays.

Data were analysed using multivariate mixed effects multilevel modelling with robust standard errors.

Factors of interest were: ethnicity, holiday/term, sex, socio-economic status (SES), weight status, weekend/weekday and season.

Results: One-hundred-and-eight children (67.5%) provided 1157 valid days of data. Fifty nine percent of children were WB (n=64) and 41% (n=44) were SA. Boys spent more time in MVPA (11 min/d, p=0.013) compared to girls and SA children spent more time in SB (39 minutes, p=0.017) compared to WB children in adjusted models. Children living in higher SES areas were more sedentary (43 minutes, p=0.006) than children living in low SES areas. Children were more active during summer (15 minutes MVPA, p<0.001; 27 LPA, p<0.001) and spring (15 minutes MVPA, p=0.005; 38 minutes LPA, p<0.001) and less sedentary (-42 minutes and -53 minutes, p<0.001) compared to winter. Less time (8 minutes, p=0.012) was spent in LPA during school terms compared to school holidays. Children spent more time in MVPA (5 minutes, p=0.036) during weekend compared to weekdays. Overweight and obese children spent more time in LPA (21 minutes, p=0.021) than normal-weight children.

Conclusion: The results of our study suggest that significant child level factors associated with movement behaviours are ethnicity, sex, weight-status and area SES. Significant temporal factors are weekends, school holidays and seasonality. Interventions to support health enhancing movement behaviours may need to be tailored around these factors.

For peer review only - http://bmjopen.bmj.com/site/about/guidelines.xhtml

Article summary

Strengths and limitations (five bullet points relating to the methods)

- First study in Europe to evaluate accelerometer-measured physical activity in school terms and school holidays
- Includes White British and South Asian children
- Participants selected from high and low socioeconomic areas
- The use of accelerometers underestimate water-based activities
- The demographics of the population might limit the generalisability of the results

Introduction

High levels of sedentary behaviour (SB) and low levels of physical activity (PA) are common among primary school children and have become a public health concern due to negatively impacting physical and mental health (1–3).

To date, studies have reported lower levels of PA and higher levels of SB in South Asian (SA) adults and children when compared to White British (WB) adults and children (4–10). Often these differences have been explained on the account of cultural preferences ,socioeconomic factors , lack of intrinsic motivation, lack of understanding about the benefits of PA and lack of understanding how to integrate PA in daily routine (11,12). Within the UK, the majority of studies in the field of child PA and SB have been conducted with WB children (13–17) and very few have considered ethnic differences between WB and South Asian (SA) children (9,18,19). The SA population accounts for nearly 8% of the UK population and the percentage rises up to 44% in various cities in the UK (20).

More recently, researchers in the field of movement behaviour have started to investigate different compositions of behaviours to identify those compositions which are most health enhancing (21–24). For example, Saunders et al. (25), investigated a reduction in light physical activity (LPA) or SB versus an increase in moderate to vigorous physical activity (MVPA) and concluded that children characterized by low SB, high PA and high levels of sleep have more desirable health outcomes (25). Public health messages are consistent with these insights and have started adopting slogans such as “Move more, sit less” to promote positive health behaviours in adults and children (23). This indicates that movement behaviour should be considered as a whole instead of focusing on PA or SB separately.

Since the SA population is at higher risk of developing diabetes and cardiovascular ill health (26–28) and as lower levels of PA and higher levels of SB contribute to increased risk of cardio-metabolic ill health (2,29)

For peer review only

For peer review only - http://bmjopen.bmj.com/site/about/guidelines.xhtml

including SA participants in studies merits further investigation as the findings may inform the design and development of interventions.

A recent observational study with over 18,000 children (30) identified overweight and obesity prevalence increase during the school holiday suggesting that there are major risk factors for overweight and obesity outside of school term. For a movement point of view, it can be hypothesized that the increase in overweight and obesity is partly due to less PA and increased SB. Although children spend about 20% of the calendar year in school holidays, few studies have considered movement behaviours during school holidays. It has been hypothesized that PA would decrease while SB would increase during the school holidays when compared to school terms (31,32) because the unstructured time in holidays is more likely to foster an increase in SB and a decrease in PA (33). Only one other study based in Japan evaluated objectively measured physical activity during school holidays and concluded that PA was higher during the school holidays(31).
A possible explanation for these results are parents' levels of SB and PA. Children tend to spend more time with their parents during school holidays; if their parents have high levels of SB and low levels of PA it is likely for this behaviour to be adopted by the children also.

The aim of this study was to investigate factors associated with objectively-measured movement behaviour among WB and SA primary school aged children in the District of Bradford, UK, during the school terms and school holidays. Bradford is the 6th largest city in the UK and the third with the highest population of under 16-year- olds (34). It is a highly deprived city and the health outcomes of the population are considerably worse compared with the rest of the UK (35,36).

Methods

Ethical approval for the study was obtained from the University of Bradford Ethical Committee (E536, 2016). Head teachers from fifteen schools were approached to take part; three volunteered and were selected. Schools were selected based on the SES and ethnicity of the children they served. Two schools had predominantly WB children (one with children of low SES and one high) and one school with predominantly SA children with low SES.
Four-hundred and ninety-two, 6- to- 8-year-old children with a mean age of 7.52 years (SD=0.5) from three schools with a Bradford postcode were invited to participate in this cross-sectional study conducted between July 2016 and May 2017. One-hundred and sixty-two children were consented to participate in the study by parents. The schools were attended by children living in postcodes of varying Indices of Multiple Deprivation (IMD) of between 1 and 8).

Data were collected at five time points: summer (July 2016 term) ; n=55 consented /152 invited), winter (January to February 2017 term and holiday; n=69/154) and spring (April to May 2017 term and holiday;

For peer review only - http://bmjopen.bmj.com/site/about/guidelines.xhtml

n=38/186). Primary school aged children were invited to wear an Actigraph GT3X+ triaxial accelerometer (Actigraph, Pensacola, FL, USA). The monitors were distributed in each school by the lead researcher. The Actigraph was attached on an elastic band around the child's waist on the right hip. Participating children were asked to wear the accelerometer during all waking hours and overnight for at least 7 days. Children who participated in the winter and spring data collections were invited to wear the device during school term and school holidays with a 1-2-week break between the two-data collection periods. Therefore, the study comprised of three groups of children according to the season of data collection: summer, winter and spring. Each child was provided with one monitor at each data collection point. Children and teachers were provided with an explanation of how the monitor worked and that they should be removed only for water activities. Written instructions were provided to parents and teachers. Prior to giving out the monitors, parents were invited to attend a presentation and discuss any concerns they might have about the study.

Procedure and measurements

Research measurements taken by trained staff included height (cm) (wall mounted stadiometer: Seca UK, Birmingham UK), weight (Kg), body composition (measured with Tanita scales TBF-300 MA, Tokyo, Japan and Tanita scales BC-418 MA, Tokyo, Japan), and waist circumference (Seca anthropomentry tape) measured just above the exposed naval. Height was recorded to the nearest 0.1 cm and 0.1 kg, with shoes and socks removed. BMI values were calculated (weight (kg)/height (m)^2) and BMI (body mass index) z-scores were calculated for each participant using the British growth reference(37). Weight categories were derived from BMI percentiles using Freeman et al. (38) recommendations.

Parents completed a demographics questionnaire which included child postcode, date of birth and ethnicity. Based on each child's home postcode, the Index of Multiple Deprivation (IMD) was determined and collapsed into three levels of SES: low (IMD1-2), medium (IMD3-5) and high (IMD>6)(39).

Data management and processing

The accelerometer was set to record data at a sampling rate of 60 Hertz. Raw accelerometer data were downloaded and collapsed into 15s epoch files. The Evenson (40) cut-points were selected for this study because they have been reported to be among of the most accurate to estimate MVPA for children aged 6-to-8-year-old (41). The calibration studies for these cut off points meet the 4 item criteria by Freedson et al. (42) and Welk et al (43). The choice for 15s epoch length was in line with the calibration studies for this population group age (40). Non-wear time was defined as ≥ 20 minutes of consecutive zero counts as they are the most reported ; 10-30 minutes is one of the most reported non-wear periods (44).

The minimum valid wear time for inclusion in the analysis was at least 10 hours on at least three-week days and at least one weekend day (Saturday or Sunday) for any data collection. This criteria of valid data inclusion is reported to have high reliability for estimates of PA (45). Sleep time was removed from the data each day

For peer review only - http://bmjopen.bmj.com/site/about/guidelines.xhtml
For peer review only

between 11 pm and 5 am similarly to other studies for primary school aged children (46,47) but also to take into consideration that the majority of SA children in our study were Muslims who are encouraged to wake up for the dawn prayer, which during spring and summer is around 5 am(48).

Patient and public involvement (PPI)

The research team regularly convenes a Born in Bradford Parent Governors PPI groups (https://borninbradford.nhs.uk/about-us/our-parents-governors/).This study was born out of parental concerns that levels of physical activity and sedentary time are high in childhood. Parents in the group were shown possible devices that could be used to measure physical activity (including Actiheart, activPAL, wrist and waist worn Actigraphs). The waist worn Actigraph was their preferred measure. Parents suggested that the best way to access children for the study was via schools; the PPI groups was not involved with recruitment either in o the study or the study conduct.

Data Analysis

Statistical analyses were conducted using Stata v.13.1 (49). The three outcome variables were SB, LPA and MVPA. Since the study design involves repeated continuous measures per child, we used a multivariate linear mixed effects multilevel modelling with child as a random effect and response variables as repeated measures nested within each child. We assumed an identity covariance structure with robust standard errors to mitigate the impact of heteroskedasticity from skewed outcome variables. We included school as a random effect, but with only three schools we found a low Intra Class Correlation (ICC) (SB ICC=0.001, LPA ICC=0.01, and MVPA ICC = 0.01) (see Table 1). School was entered as a fixed effect covariate. All models were adjusted for the following factors: wear time, ethnicity, holiday/term, sex, SES, weight status, weekend/weekday, season and school. P-values of < 0.05 were defined as statistically significant. Model coefficients are displayed as mean (± SD) and mean (95% Confidence Intervals (CI)) in the text and tables.

For peer review only - http://bmjopen.bmj.com/site/about/guidelines.xhtml

Table 1: Performance of unconditional and full models for each outcome SB, LPA, and MVPA.

**Models**	**SB**	**LPA**	**MVPA**
Nested vs. non-nested Likelihood ratio test	246.02 (p<0.001)	345.72 (p<0.001)	393.15 (p<0.001)
Unconditional models (school as a random effect)			
ICC	0.001	0.01	0.01
AIC	13991.08	12820.21	11379.40
BIC	14006.24	12835.37	11394.56
Log-likelihood	-6992.54	-6407.10	-5686.70
Unconditional models (child as a random effect)			
ICC	0.33	0.39	0.45
AIC	13745.28	12482.51	10996.79
BIC	13760.44	12497.67	11011.95
Log-likelihood	-6869.64	-6238.26	-5495.40
Full model (child as a random effect)			
ICC	0.33	0.35	0.37
AIC	12807.72	12097.39	10950.04
BIC	12.883.53	12173.19	11025.85
Log-likelihood	-6388.86	-6033.70	-5460.02

<u>Results</u>

Out of 492 invited children, consent and assent was available for 160 (32.5%). A further 54 children were excluded because they did not meet the inclusion criteria for valid data leaving 108 (67.5%, 108/160) children who had valid data included in the analyses.

Table 2 shows the profile of the children. The mean age of the 108 children was 7.5 years with 51% girls; 59% (64/108) WB and 41% (44/108) were SA. Forty-one (38%; 41/108) children were from the 20% most deprived areas (IMD 1-2), however that was the case for the majority of SA (80%; 35/44) children. About three-quarters of children (77%, 83/108) were of normal weight and 23% (25/108) were overweight and obese although a higher percentage (27%; 12/44) of SA than WB children were overweight and obese (Table 2).

When we compared the characteristics of the children whose data was included in the analyses to those excluded (n=54), we did not find any differences relating to age, sex, SES or overweight status.

For peer review only - http://bmjopen.bmj.com/site/about/guidelines.xhtml

Table 2: Characteristics of children whose data was included in the analyses.

Characteristic	White British N=64 (59%)	South Asian N=44 (41%)	All children N=108
Age (years), mean (sd)	7.52(0.50)	7.61 (0.49)	7.52 (0.50)
Male (%)	32 (50)	21 (48)	53 (49)
Female (%)	32 (50)	23 (52)	55(51)
Low SES (%)	6 (9.38)	35 (79.55)	41 (38)
Medium SES (%)	40(62.50)	9 (20.35)	49 (45)
High SES (%)	18 (28.13)	0 (0.0)	18 (17)
z-BMI, mean (sd)	0.31 (.98)	0.38 (1.34)	0.34(1.14)
Normal weight (%)	51 (80)	32 (73)	83 (77)
Overweight (%)	9(14)	3 (7)	12 (11)
Obese (%)	4(6)	9 (20)	13 (12)

The 108 children who had valid data provided a total of 1157 term days and holiday days. Children (SA and WB combined) had on average 901 minutes of daily valid wear of which 539 minutes (60%) were spent SB, 296 minutes (33%) in LPA and 67 minutes (7%) were MVPA.

Table 3 gives an overview of the unadjusted wear times, SB, LPA and MVPA average minutes stratified by ethnicity, school terms and school holidays. The unadjusted results in the combined data set identified slightly higher proportions of SB (61%; 550 minutes) and LPA (33%; 292 minutes) but lower proportions of MVPA (6%; 57 minutes) for SA children compared to WB children. A similar pattern was observed when stratifying the results by term and holiday (Table 3).

For peer review only

Table 3: Unadjusted data sets averages by ethnicity.

	Combined data				School terms				School holidays			
Combined data set	WB N=64 Mean (sd)	% of wear time	SA N=34 Mean (sd)	% of wear time	WB N=56 Mean (sd)	% of wear time	SA N=39 Mean (sd)	% of wear time	WB N=40 Mean (sd)	% of wear time	SA N=23 Mean (sd)	%of wear time
Total number of valid days	684	n/a	473	-	369	n/a	269	-	315	n/a	214	-
Valid wear time in minutes	888.78 (117.16)	n/a	899.85 (110.55)	-	876.66 (105.60)	n/a	902.12 (120.55)	-	885.88 (105.83)	n/a	915.03 (85.22)	-
Total SB in minutes	532.05 (109.13)	60	550.13 (117.57)	61	532.42 (104.44)	61	553.05 (113.56)	61	522.10 (110.2)	60	564.55 (76.89)	61
Total LPA	288.73 (66.64)	32	292.27 (52.02)	33	275.54 (64.78)	31	289.27 (55.03)	32	299.90 (67.14)	33	296.90 (56.47)	32
Total MVPA	67.99 (33)	8	57.44 (37.97)	6	68.95 (31.49)	8	59.80 (34.08)	7	63.85 (31.44)	7	53.57 (24.88)	7

For peer review only

For peer review only - http://bmjopen.bmj.com/site/about/guidelines.xhtml

Table 4 reports findings from statistical models for each outcome variable: SB, LPA and MVPA following mutual adjustment for predictors of interest. Once wear time, term, ethnicity, SES, school, season, sex, weekend and weight status were mutually adjusted for, significant differences were identified for SB in relation to 3 factors: ethnicity, SES and seasonality; for LPA in relation to 4 factors: SES, seasonality, term, and overweight; and for MVPA in relation to 3 factors: sex, seasonality and weekend (Table 4).

Specifically, boys spent significantly more time in MVPA (11 min/d) compared to girls and SA children spent more time in SB (39 minutes) compared to WB children (p=0.017). Children residing in high SES area had approximately 43 min/d more sedentary time and 31 min/d less LPA than children from low SES areas (p<0.001;p=0.011). Children were more active (15 minutes MVPA, 27 minutes LPA) and less sedentary (-42 minutes) during summer and spring (15 minutes MVPA, 38 minutes LPA, -53 minutes SB) compared to winter (p<0.001).Less time in LPA (9 minutes) during school terms compared to school holidays (p=0.012). Children spent more time in MVPA (5 minutes) during weekends compared to weekdays (p=0.036). Overweight and obese children spent more time in LPA (21 minutes) than normal weight children (p=0.021).

For peer review only - http://bmjopen.bmj.com/site/about/guidelines.xhtml
For peer review only

Table 4: Model coefficients for three movement behaviour outcomes based on the combined data set (term and holiday days; n=108) (CI 95%).

Covariates	SB<br>Coefficient, CI, p-value	LPA<br>Coefficient, CI, p-value	MVPA<br>Coefficient, CI, p-value
Total Wear time	0.68<br>(0.64 to 0.71)<br>p <0.001	0.28<br>(0.25 to 0.31)<br>p <0.001	0.05<br>(0.03 to 0.06)<br>p <0.001
South Asian	38.51<br>(6.79 to 70.23)<br>p = 0.017	-23.66<br>(-47.46 to 0.15)<br>p = 0.051	-14.94<br>(-34.41 to 4.54)<br>p = 0.133
Male	-1.15<br>(-18.88 to 16.59)<br>p = 0.899	-9.87<br>(-23.08 to 3.33)<br>p = 0.143	11.03<br>(2.33 to 19.72)<br>p = 0.013
Medium SES<br>(ref Low SES)	15.82<br>(-6.09 to 37.72) p =<br>0.157	-17.29<br>(-31.65 to -2.92)<br>p = 0.018	1.65<br>(-12 to 15.3)<br>p = 0.813
High SES<br>(ref Low SES)	43.26<br>(12.35 to 74.18)<br>p = 0.006	-31.28<br>(-55.36 to -7.2)<br>p = 0.011	-11.85<br>(-27.61 to 3.9)<br>p = 0.14
School A (ref school B)	18<br>(-23.86 to 59.86)<br>p = 0.399	-14.01<br>(-45.87 to 17.85)<br>p = 0.389	-4.31<br>(-28.37 to 19.74)<br>p = 0.725
School C (ref school B)	18.64<br>(-14.36 to 51.65)<br>p = 0.268	-11.37<br>(-36.68 to 13.95)<br>p = 0.379	-7.36<br>(-27.06 to 12.35)<br>p = 0.464
Spring (ref Winter)	-53.48<br>(-81.56 to -25.4)<br>p<0.001	38.07<br>(17 to 59.13)<br>p <0.001	15.48<br>(0 to 30.97)<br>p =0.05
Summer (ref winter	-42.44<br>(-59.61 to -25.28)<br>p < 0.001	26.99<br>(13.28 to 40.7)<br>p < 0.001	15.47<br>(7.15 to 23.8)<br>p <0.001
Term (ref holiday)	8.04<br>(-1.38 to 17.46)<br>p = 0.094	-8.64<br>(-15.38 to -1.9)<br>p = 0.012	0.59<br>(-3.84 to 5.01)<br>p = 0.796
Weekend (ref weekday)	-6.02<br>(-15.44 to 3.4)<br>p = 0.211	1.39<br>(-5.66 to 8.43)<br>p = 0.7	4.67<br>(0.31 to 9.02)<br>p = 0.036
Overweight and Obese<br>(ref Normal weight)	-19.18<br>(-42.03 to 3.68)<br>p = 0.1	21.13<br>(3.23 to 39.04)<br>p = 0.021	-1.94<br>(-11.25 to 7.37)<br>p = 0.683
Intercept	-86.66<br>(-133.21 to -40.1)<br>p < 0.001	64.81<br>(29.53 to 100.09)<br>p < 0.001	21.7<br>(-1.16 to 44.55)<br>p = 0.063
Variance of level-2<br>(child)	147.11	915.14	357.36
ICC	0.33	0.35	0.37

SB=Sedentary behaviour; LPA=Light physical activity; MVPA =Moderate to vigorous physical activity

For peer review only

Discussion

Summary of findings

Overall, children spent a large proportion of time in SB, approximately 60-61% (522 to 564 min/d), followed by LPA, approximately 31-33% (276 to 300 minutes) and a smaller proportion in MVPA, 6-8 % (54 to 69 minutes. We also identified other key findings: 1) SA children were more sedentary than WB children (2) boys were more active than girls; (3) There were no significant differences for SB and MVPA, yet LPA was lower during term compared to school holiday; 4) Children had more MVPA during weekends compared to weekdays; 5) Children were markedly more active and less sedentary during the summer and spring compared to winter; 5) Overweight and obese children has more LPA than normal weight children and 6) children from high SES were more sedentary and less active (LPA) compared to children from low SES.

Comparison to other studies

We found that SA children spent 39 more min/d in SB compared to WB children which is similar to the results of other studies evaluating ethnic differences in PA and SB in WB and SA , 8 to 9 year old children (50) and 9 to 11 year old girls in England(10). Both studies found that SA children were less active and more sedentary compared to WB children. The results of the Millennium study also reported that SA children were the most sedentary and least active when compared to WB or children of African origin (51).

One possible explanation for higher SB in SA children is Mosque attendance for religious education and Arabic language. Data from 2356 children of SA ethnicity from the Born in Bradford cohort study identified that 71 % of SA children attended the religious setting most days of the week and spent 1-2 hours here. It is likely that the time spent here is seated and this might explain higher sedentary time for SA children. Furthermore, it has previously been shown that SA children watch more TV from a younger age (9)and sleep less overnight allowing more time for SB (52). Results from the Born in Bradford study have also shown that both sleep and TV- viewing are also related to fatness/ obesity in Bradford children (higher in SA than in WB) which is inversely associated with PA levels (8).

Overall, children spent a large proportion of time in SB, followed by LPA, and a smaller proportion in MVPA, 6-8 % (57 to 69 minutes) (Table 3). Although the children in our study had high levels of SB, the average level of MVPA was higher than compared with other studies (51,53). Results from the Millennium Study(51) on 13000, 7 year of children identified an average of 38 minutes/ day spent in MVPA. The Millennium study however had a different definition of a valid day and included days with >6 hours or valid wear time. It is possible that the higher levels of MVPA in our study are related data being collected over three weeks of summer and spring and only two weeks of winter.

For peer review only - http://bmjopen.bmj.com/site/about/guidelines.xhtml

There were only modest differences between the school term and the school holidays, with 8.64 fewer min/d of LPA spent during the school term time compared to the school holidays. Our finding lower LPA and no differences for SB and MVPA during the school term is contrary to previous reports of higher SB and lower PA during school holidays (54). It is possible that children maintain the movement habits from the school term during the school holidays despite the lack of unstructured time for some and despite not needing to sit down for lessons. Currently the majority of interventions aimed at increasing PA and/or reducing SB focuses on school term time only (55–57). Our results imply that interventions to promote positive movement behaviour are needed also during holidays as high levels of SB have been recorded during both occasions. From a practical point of view for data collection and interventions, children are more accessible en-masse during the school term, however the school holiday period should be considered as it appears to be a critical time period for some children. In a recent meta-analysis (58) which highlighted reasons for the lack of effectiveness of PA interventions it was suggested that the positive outcomes of PA interventions during school hours are not maintained outside of school hours and that interventions should include homes and communities.

Seasonality was identified as a consistent correlate of all movement behaviours (SB lower and LPA, MVPA higher during the summer and spring season compared to winter). These patterns are similar to the study of Atkin et al (59) which identified elevated levels of SB in winter and autumn for children aged 7-years and suggested the need for interventions during autumn and winter. Similarly, a study involving over 1000 primary school aged Danish children, (53) found higher SB and lower MVPA during winter compared to spring. More studies should consider designs that allow seasonal comparisons within the same children measured repeatedly throughout the course of a year. Regarding weekdays and weekend, unlike our study, Hjorth et al. (53) identified higher SB and lower MVPA at the weekend. Most studies evaluating physical evaluating movement behaviour in children (14,19,60) have identified lower MVPA at the weekend which is contrary to our results.

Compared to children from low SES areas, children residing higher SES areas (IMD 6-8) were more sedentary and less active which is contrary to majority of the existing evidence. A possible explanation would be that children residing in higher SES area might have access to more items technology and engage in more screen viewing activities. Also, contrary to existing evidence(8,52), the overweight and obese children were more active than normal weight children .

Strengths and limitations

The strength our study is related to uniqueness of the sample which included SA and WB children from SES areas with IMD 1 to 8, and we also included data during the school holidays. To our knowledge this is the first accelerometry study in Europe to take into consideration school holidays.

For peer review only - http://bmjopen.bmj.com/site/about/guidelines.xhtml

The 33% response rate in our study is similar to response rates in similar studies taking part in more affluent areas in the UK (14,61).The sample of the study was modest which means that we may have been underpowered to detect small associations. However, our sample size was similar to a Japanese study conducted by Tanaka and colleagues which considered the summer holidays accelerometry and included 98 children in the data analysis(31). A larger study which considered PA and SB during school holidays was conducted in the USA and included 406 children but the data were collected over 20 months and PA and SB were self-reported(32).

Despite aiming to include children from various SES areas, this was challenging because the majority of SA children in Bradford were from the poorest areas with only 20% of SA children from medium SES and 0% from high SES. The use of area SES has its own limitations by introducing misclassification error(62).
Ideally a comparator school with predominantly SA children with high SES would also have been included, but the demographics of Bradford precluded this.

Another limitation of the study could be the definition of sleep and the 5 am awake time. It is possible that some sleeping hours may have been misclassified as SB, thereby overestimating SB. Our choice for the 5am awake time was made in the context of SA children, majority of Muslim faith, who are encouraged to wake up around this time for the morning prayer.

Conclusion

The results of our study suggest that children spent high proportions of their time in SB. Factors associated with movement behaviours included ethnicity, sex, weight status, area level SES and temporal factors: weekend, school holidays and seasonality. SA children were more sedentary and less active than WB children. Interventions to reduce SB and increase PA are needed over school term and school holidays. Interventions aimed at reducing SB and increasing PA are needed across all seasons but especially during the winter when SB is higher and PA is lower.

For peer review only - http://bmjopen.bmj.com/site/about/guidelines.xhtml

References:

1. LeBlanc AG, Spence JC, Carson V, Connor Gorber S, Dillman C, Janssen I, et al. Systematic review of sedentary behaviour and health indicators in the early years (aged 0–4 years). Appl Physiol Nutr Metab. 2012;37(4):753–72.

2. Tremblay M, LeBlanc A, Kho M, Saunders T, Larouche R, Colley R, et al. Systematic review of sedentary behaviour and health indicators in school-aged children and youth. Int J Behav Nutr Phys Act. 2011;8(1):98–119.

3. Hancox RJ, Milne BJ, Poulton R. Association between child and adolescent television viewing and health: A longitudinal birth cohort study. Lancet [Internet]. 2004;364(9430):257–62. Available from: http://search.proquest.com.ezproxy.library.yorku.ca/docview/620529140?accountid=15182%5Cnhttp://sfx.scholarsportal.info/york?url_ver=Z39.88-2004&rft_val_fmt=info:ofi/fmt:kev:mtx:journal&genre=article&sid=ProQ:ProQ:psycinfo&atitle=Association+between+child

4. Khunti K, Stone MA, Bankart J, Sinfield PK, Talbot D, Farooqi A, et al. Physical activity and sedentary behaviours of South Asian and white European children in inner city secondary schools in the UK. Fam Pract. 2007;24(3):237–44.

5. Fischbacher CM, Hunt S, Alexander L. How physically active are South Asians in the United Kingdom? A literature review. Journal of Public Health. 2004.

6. Williams ED, Stamatakis E, Chandola T, Hamer M. Physical activity behaviour and coronary heart disease mortality among South Asian people in the UK: An observational longitudinal study. Heart. 2011;

7. Sherry AP, Pearson N, Ridgers ND, Barber SE, Bingham DD, Nagy LC, et al. activPAL-measured sitting levels and patterns in 9–10 years old children from a UK city. J Public Health (Bangkok) [Internet]. 2018 Oct 20;fdy181-fdy181. Available from: http://dx.doi.org/10.1093/pubmed/fdy181

8. Collings PJ, Brage S, Bingham DD, Costa S, West J, McEachan RRC, et al. Physical Activity, Sedentary Time, and Fatness in a Biethnic Sample of Young Children. Med Sci Sports Exerc [Internet]. 2017 May;49(5):930–8. Available from: http://www.ncbi.nlm.nih.gov/pmc/articles/PMC5392414/

9. Barber SE, Kelly B, Collings PJ, Nagy L, Bywater T, Wright J. Prevalence , trajectories , and determinants of television viewing time in an ethnically diverse sample of young children from the UK. Int J Behav Nutr Phys Act. 2017;14:88.

10. Pollard TM, Hornby-Turner YC, Ghurbhurrun A, Ridgers ND. Differences between 9-11 year old British Pakistani and White British girls in physical activity and behavior during school recess. BMC Public Health [Internet]. BMC Public Health; 2012;12(1):1087. Available from: http://www.pubmedcentral.nih.gov/articlerender.fcgi?artid=3551783&tool=pmcentrez&rendertype=abstract

For peer review only - http://bmjopen.bmj.com/site/about/guidelines.xhtml

11. Jepson R, Harris FM, Bowes A, Robertson R, Avan G, Sheikh A. Physical activity in South Asians: an in-depth qualitative study to explore motivations and facilitators. PLoS One. 2012;

12. Nazroo JY. The Structuring of Ethnic Inequalities in Health: Economic Position, Racial Discrimination, and Racism. Am J Public Health [Internet]. © American Journal of Public Health 2003; 2003 Feb 8;93(2):277–84. Available from: http://www.ncbi.nlm.nih.gov/pmc/articles/PMC1447729/

13. King AC, Parkinson KN, Adamson AJ, Murray L, Besson H, Reilly JJ, et al. Correlates of objectively measured physical activity and sedentary behaviour in English children. Eur J Public Health. 2011;21(4):424–31.

14. Collings PJ, Wijndaele K, Corder K, Westgate K, Ridgway CL, Dunn V, et al. Levels and patterns of objectively-measured physical activity volume and intensity distribution in UK adolescents: the ROOTS study. Int J Behav Nutr Phys Act [Internet]. 2014;11(1):23. Available from: http://ijbnpa.biomedcentral.com/articles/10.1186/1479-5868-11-23

15. Chinapaw MJM, Proper KI, Brug J, van Mechelen W, Singh AS. Relationship between young peoples' sedentary behaviour and biomedical health indicators: A systematic review of prospective studies. Obes Rev. 2011;12(7).

16. Nelson MC, Neumark-Stzainer D, Hannan PJ, Sirard JR, Story M. Longitudinal and secular trends in physical activity and sedentary behavior during adolescence. Pediatrics [Internet]. 2006;118. Available from: https://doi.org/10.1542/peds.2006-0926

17. Townsend N, Wickramasinghe K, Williams J, Bhatnagar P, Rayner M. Physical activity statistics. British Heart Foundation. 2015. 1-128 p.

18. Hornby-Turner YC, Hampshire KR, Pollard TM. A comparison of physical activity and sedentary behaviour in 9-11 year old British Pakistani and White British girls: A mixed methods study. Int J Behav Nutr Phys Act [Internet]. 2014;11(1):74. Available from: http://www.pubmedcentral.nih.gov/articlerender.fcgi?artid=4059029&tool=pmcentrez&rendertype=abstract

19. Eyre ELJ, Duncan MJ, Smith EC, Matyka KA. Objectively measured patterns of physical activity in primary school children in Coventry: the influence of ethnicity. Diabet Med. England; 2013 Aug;30(8):939–45.

20. Office for National Statistics. Census 2011. Census [Internet]. 2011;(May):78. Available from: http://www.ons.gov.uk/ons/guide-method/census/2011/index.html

21. Ekelund U, Luan J, Sherar LB, Esliger DW, Griew P, Cooper A. Moderate to vigorous physical activity and sedentary time and cardiometabolic risk factors in children and adolescents. JAMA [Internet]. 2012;307. Available from: https://doi.org/10.1001/jama.2012.156

22. Tremblay MS, Carson V, Chaput J-P, Connor Gorber S, Dinh T, Duggan M, et al. Canadian 24-Hour Movement Guidelines for Children and Youth: An Integration of Physical Activity, Sedentary Behaviour, and Sleep. Appl Physiol Nutr Metab. 2016;41(6 (Suppl. 3)).

For peer review only - http://bmjopen.bmj.com/site/about/guidelines.xhtml

23. Dumuid D, Pedišić Ž, Stanford TE, Martín-Fernández J-A, Hron K, Maher CA, et al. The compositional isotemporal substitution model: A method for estimating changes in a health outcome for reallocation of time between sleep, physical activity and sedentary behaviour. Stat Methods Med Res [Internet]. SAGE Publications Ltd STM; 2017 Nov 20;0962280217737805. Available from: https://doi.org/10.1177/0962280217737805

24. Pedišić Ž, Dumuid D, Olds TS. Integrating sleep, sedentary behaviour, and physical activity research in the emerging field of time-use epidemiology: Definitions, concepts, statistical methods, theoretical framework, and future directions. Kinesiology. 2017;49(2):135–45.

25. John Saunders T, Ellen Gray C, Joan Poitras V, Chaput J-P, Janssen I, Katzmarzyk PT, et al. Combinations of physical activity, sedentary behaviour and sleep: relationships with health indicators in school-aged children and youth 1. Appl Physiol Nutr Metab Appl Physiol Nutr Metab Downloaded from [Internet]. 2016;41(June):283–93. Available from: www.nrcresearchpress.com

26. H.M. M, N. C, J.H. F. Mortality and morbidity from diabetes in South Asians and Europeans: 11-year follow-up of the Southall Diabetes Survey, London, UK. Diabet Med [Internet]. Wiley-Blackwell; 2004 Jul 19;15(1):53–9. Available from: https://doi.org/10.1002/(SICI)1096-9136(199801)15:1%3C53::AID-DIA521%3E3.0.CO

27. Gholap N, Davies M, Patel K, Sattar N, Khunti K. Type 2 diabetes and cardiovascular disease in South Asians. Prim Care Diabetes [Internet]. Elsevier; 2011 Apr 1;5(1):45–56. Available from: http://dx.doi.org/10.1016/j.pcd.2010.08.002

28. Bhopal R, Hayes L, White M, Unwin N, Harland J, Ayis S, et al. Ethnic and socio-economic inequalities in coronary heart disease, diabetes and risk factors in Europeans and South Asians. J Public Health (Bangkok) [Internet]. 2002 Jun 1;24(2):95–105. Available from: http://dx.doi.org/10.1093/pubmed/24.2.95

29. Chastin SFM, Palarea-Albaladejo J, Dontje ML, Skelton DA. Combined effects of time spent in physical activity, sedentary behaviors and sleep on obesity and cardio-metabolic health markers: A novel compositional data analysis approach. PLoS One. 2015;10(10).

30. von Hippel PT, Workman J. From Kindergarten Through Second Grade, U.S. Children's Obesity Prevalence Grows Only During Summer Vacations. Obesity. 2016;

31. Tanaka C, Reilly JJ, Tanaka M, Tanaka S. Seasonal changes in objectively measured sedentary behavior and physical activity in Japanese primary school children. BMC Public Health [Internet]. London: BioMed Central; 2016 Sep 13;16(1):969. Available from: http://www.ncbi.nlm.nih.gov/pmc/articles/PMC5020446/

32. Staiano AE, Broyles ST, Katzmarzyk PT. School Term vs. School Holiday: Associations with Children's Physical Activity, Screen-Time, Diet and Sleep. Tchounwou PB, editor. Int J Environ Res Public Health [Internet]. MDPI; 2015 Aug 30;12(8):8861–70. Available from: http://www.ncbi.nlm.nih.gov/pmc/articles/PMC4555252/

For peer review only

For peer review only - http://bmjopen.bmj.com/site/about/guidelines.xhtml

33. Arundell L, Fletcher E, Salmon J, Veitch J, Hinkley T. A systematic review of the prevalence of sedentary behavior during the after-school period among children aged 5-18 years. Int J Behav Nutr Phys Act [Internet]. International Journal of Behavioral Nutrition and Physical Activity; 2016 Aug;13(1):93. Available from: http://ijbnpa.biomedcentral.com/articles/10.1186/s12966-016-0419-1
34. ONS. Census 2011. Census [Internet]. 2011;(May):78. Available from: http://www.ons.gov.uk/ons/guide-method/census/2011/index.html
35. Firth C, Petherick E, Oddie SJ. Infant deaths from congenital anomalies: novel use of Child Death Overview Panel data. Arch Dis Child [Internet]. 2018;archdischild-2017-314256. Available from: http://www.ncbi.nlm.nih.gov/pubmed/29436407%0Ahttps://www.ncbi.nlm.nih.gov/pubmed/29436407
36. Wright J, Small N, Raynor P, Tuffnell D, Bhopal R, Cameron N, et al. Cohort profile: The born in bradford multi-ethnic family cohort study. Int J Epidemiol. 2012;42(4):978–91.
37. Cole TJ, Freeman J V, Preece MA. Body mass index reference curves for the UK, 1990. Arch Dis ChildhoodArch Dis Child. 1995;73(73):25–9.
38. Freeman V, Cole TJ, Chinn S, Jones PRM, White EM, Preece MA. Cross sectional stature and weight reference curves for the UK , 1990. 1995;17–24.
39. ONS. The English Indices of Deprivation 2015 Statistical Release. 2015.
40. Evenson KR, Catellier DJ, Gill K, Ondrak KS, McMurray RG. Calibration of two objective measures of physical activity for children. J Sports Sci. 2008;26(14):1557–65.
41. Kim Y, Lee J-M, Peters BP, Gaesser GA, Welk GJ. Examination of different accelerometer cut-points for assessing sedentary behaviors in children. PLoS One [Internet]. Department of Kinesiology, Iowa State University, Ames, Iowa, United States of America. School of Health, Physical Education and Recreation College of Education, University of Nebraska, Omaha, Nebraska, United States of America. School of Nutrition and He; 2014;9(4):e90630–e90630. Available from: http://search.ebscohost.com/login.aspx?direct=true&db=cmedm&AN=24699259&site=ehost-live
42. Freedson P, Pober D, Janz KF. Calibration of accelerometer output for children. In: Medicine and Science in Sports and Exercise. 2005.
43. Welk GJ. Principles of design and analyses for the calibration of accelerometry-based activity monitors. In: Medicine and Science in Sports and Exercise. 2005.
44. Aadland E, Andersen LB, Anderssen SA, Resaland GK. A comparison of 10 accelerometer non-wear time criteria and logbooks in children. BMC Public Health [Internet]. London: BioMed Central; 2018 Mar 6;18:323. Available from: http://www.ncbi.nlm.nih.gov/pmc/articles/PMC5840816/
45. Rich C, Geraci M, Griffiths L, Sera F, Dezateux C, Cortina-Borja M. Quality Control Methods in Accelerometer Data Processing: Defining Minimum Wear Time. PLoS One. 2013;8(6):1–8.
46. Sherry A, Pearson N, Ridgers ND, Barber SE, Bingham DD, Nagy LC, et al. Inclinometer measured sitting patterns in 9-10 year old chidlren from a northern UK city. Eur J Public. 2018;in press.

For peer review only

For peer review only - http://bmjopen.bmj.com/site/about/guidelines.xhtml

47. Meredith-Jones K, Williams S, Galland B, Kennedy G, Taylor R. 24 h Accelerometry: impact of sleep-screening methods on estimates of sedentary behaviour and physical activity while awake. J Sport Sci. 2015;0414(March).

48. BaHammam AS, Gozal D. Qur'anic insights into sleep. Nat Sci Sleep [Internet]. Dove Medical Press; 2012 Jul 24;4:81–7. Available from: http://www.ncbi.nlm.nih.gov/pmc/articles/PMC3630974/

49. StataCorp. Stata Statistical Software: Release 13. 2013. 2013.

50. E.L.J. E, M.J. D, S.L. B, V.M. C. Low socio-economic environmental determinants of children's physical activity in Coventry, UK: A Qualitative study in parents. Prev Med Reports [Internet]. E.L.J. Eyre, Department of Applied Science and Health, Coventry University, Coventry, United Kingdom; 2014;1:32–42. Available from: http://www.embase.com/search/results?subaction=viewrecord&from=export&id=L602213615

51. Griffiths LJ, Pouliou T, Rich C, Geraci M, Cortina-Borja M, Sera F, et al. Objectively measured physical activity and sedentary behaviour in UK children of primary school age: a longitudinal cohort study. Lancet [Internet]. Elsevier; 2012 Nov 23;380:S44. Available from: http://dx.doi.org/10.1016/S0140-6736(13)60400-6

52. Collings PJ, Kelly B, West J, Wright J. Associations of TV Viewing Duration, Meals and Snacks Eaten When Watching TV, and a TV in the Bedroom with Child Adiposity. Obesity. 2018;26(10):1619–28.

53. Hjorth MF, Chaput J-P, Michaelsen K, Astrup A, Tetens I, Sjödin A. Seasonal variation in objectively measured physical activity, sedentary time, cardio-respiratory fitness and sleep duration among 8-11 year-old Danish children: a repeated-measures study. BMC Public Health [Internet]. 2013;13(1):808. Available from: http://bmcpublichealth.biomedcentral.com/articles/10.1186/1471-2458-13-808

54. Brazendale K, Beets MW, Weaver RG, Pate RR, Turner-mcgrievy GM, Kaczynski AT, et al. Understanding differences between summer vs . school obesogenic behaviors of children : the structured days hypothesis. International Journal of Behavioral Nutrition and Physical Activity; 2017;1–14.

55. Atkin AJ, Gorely T, Biddle SJH, Cavill N, Foster C. Interventions to Promote Physical Activity in Young People Conducted in the Hours Immediately After School : A Systematic Review. 2011;176–87.

56. O'Dwyer M V., Fairclough SJ, Ridgers ND, Knowles ZR, Foweather L, Stratton G. Effect of a school-based active play intervention on sedentary time and physical activity in preschool children. Health Educ Res. 2013;28(6).

57. Sluijs EM, McMinn AM, Griffin SJ. Effectiveness of interventions to promote physical activity in children and adolescents: systematic review of controlled trials. BMJ [Internet]. 2007;335. Available from: https://doi.org/10.1136/bmj.39320.843947.BE

58. Love R, Adams J, van Sluijs EMF. Are school-based physical activity interventions effective and equitable? A systematic review and meta-analysis of cluster randomised controlled trials. Lancet

For peer review only - http://bmjopen.bmj.com/site/about/guidelines.xhtml

[Internet]. Elsevier; 2018 Nov 1;392:S53. Available from: https://doi.org/10.1016/S0140-6736(18)32174-3

59. Atkin AJ, Shard SJ, O HFL, Brage S, Van Sluijs EMF. Seasonal Variation in Children's Physical Activity and Sedentary Time. Med Sci Sports Exerc [Internet]. Lippincott Williams & Wilkins; 2016 Mar 18;48(3):449–56. Available from: http://www.ncbi.nlm.nih.gov/pmc/articles/PMC4762193/

60. Fairclough SJ, Boddy LM, Mackintosh KA, Valencia-Peris A, Ramirez-Rico E. Weekday and weekend sedentary time and physical activity in differentially active children. J Sci Med Sport. 2015;18(4):444–9.

61. Keevil VL, Cooper AJM, Wijndaele K, Luben R, Wareham NJ, Brage S, et al. Objective Sedentary Time, Moderate-to-Vigorous Physical Activity, and Physical Capability in a British Cohort. Med Sci Sports Exerc. 2016;48(3):421–9.

62. Stalsberg R, Pedersen A V. Effects of socioeconomic status on the physical activity in adolescents: A systematic review of the evidence. Scandinavian Journal of Medicine and Science in Sports. 2010.

For peer review only

For peer review only - http://bmjopen.bmj.com/site/about/guidelines.xhtml

Funding Statement:

This research was funded by the NIHR Collaboration for Leadership in Applied Health Research and Care Yorkshire and Humber (NIHR CLAHRC YH). www.clahrc-yh.nihr.ac.uk. PJC is funded by a BHF Immediate Postdoctoral Basic Science Research Fellowship (FS/17/37/32937). The views and opinions expressed are those of the authors, and not necessarily those of the NHS, the NIHR or the Department of Health, or any other funding agency.

Competing interest statement:

None.

Authors' contribution:

Study design and research question: LCN, MH, SEB, MAM, and PJC.

Data collection: LCN, MH, PJC.

Data processing and analysis: All authors contributed to discussions on data analysis. Data processing and analysis was carried out by LCN, MAM and MF.

Ongoing discussion and comments: All

Drafted paper and final paper: LCN

Patient consent form:

N/A

Data sharing statement:

Data is not available to the public due to participant consent restrictions.

Acknowledgements

We thank all the participants and their families for their time and ongoing participation in the study. We also thank all the researchers involved in data collection.

For peer review only

For peer review only - http://bmjopen.bmj.com/site/about/guidelines.xhtml

STROBE Statement—checklist of items that should be included in reports of observational studies

	Item No	Recommendation	Page No.
**Title and abstract**	1	(*a*) Indicate the study's design with a commonly used term in the title or the abstract	2
		(*b*) Provide in the abstract an informative and balanced summary of what was done and what was found	2
**Introduction**			
Background/rationale	2	Explain the scientific background and rationale for the investigation being reported	3-4
Objectives	3	State specific objectives, including any prespecified hypotheses	4
**Methods**			
Study design	4	Present key elements of study design early in the paper	4-5
Setting	5	Describe the setting, locations, and relevant dates, including periods of recruitment, exposure, follow-up, and data collection	4-5
Participants	6	(*a*) *Cohort study*—Give the eligibility criteria, and the sources and methods of selection of participants. Describe methods of follow-up<br>*Case-control study*—Give the eligibility criteria, and the sources and methods of case ascertainment and control selection. Give the rationale for the choice of cases and controls<br>*Cross-sectional study*—Give the eligibility criteria, and the sources and methods of selection of participants	4
		(*b*) *Cohort study*—For matched studies, give matching criteria and number of exposed and unexposed<br>*Case-control study*—For matched studies, give matching criteria and the number of controls per case	
Variables	7	Clearly define all outcomes, exposures, predictors, potential confounders, and effect modifiers. Give diagnostic criteria, if applicable	7
Data sources/ measurement	8*	For each variable of interest, give sources of data and details of methods of assessment (measurement). Describe comparability of assessment methods if there is more than one group	7
Bias	9	Describe any efforts to address potential sources of bias	7
Study size	10	Explain how the study size was arrived at	7
Quantitative variables	11	Explain how quantitative variables were handled in the analyses. If applicable, describe which groupings were chosen and why	
Statistical methods	12	(*a*) Describe all statistical methods, including those used to control for confounding	6
		(*b*) Describe any methods used to examine subgroups and interactions	
		(*c*) Explain how missing data were addressed	
		(*d*) *Cohort study*—If applicable, explain how loss to follow-up was addressed<br>*Case-control study*—If applicable, explain how matching of cases and controls was addressed<br>*Cross-sectional study*—If applicable, describe analytical methods taking account of sampling strategy	
		(*e*) Describe any sensitivity analyses	

For peer review only - http://bmjopen.bmj.com/site/about/guidelines.xhtml

**Results**			
Participants	13*	(a) Report numbers of individuals at each stage of study—eg numbers potentially eligible, examined for eligibility, confirmed eligible, included in the study, completing follow-up, and analysed	4 and7
		(b) Give reasons for non-participation at each stage	
		(c) Consider use of a flow diagram	
Descriptive data	14*	(a) Give characteristics of study participants (eg demographic, clinical, social) and information on exposures and potential confounders	7-8
		(b) Indicate number of participants with missing data for each variable of interest	
		(c) *Cohort study*—Summarise follow-up time (eg, average and total amount)	
Outcome data	15*	*Cohort study*—Report numbers of outcome events or summary measures over time	
		Case-control study—Report numbers in each exposure category, or summary measures of exposure	
		Cross-sectional study—Report numbers of outcome events or summary measures	7-11
Main results	16	(*a*) Give unadjusted estimates and, if applicable, confounder-adjusted estimates and their precision (eg, 95% confidence interval). Make clear which confounders were adjusted for and why they were included	7-11
		(*b*) Report category boundaries when continuous variables were categorized	
		(*c*) If relevant, consider translating estimates of relative risk into absolute risk for a meaningful time period	
Other analyses	17	Report other analyses done—eg analyses of subgroups and interactions, and sensitivity analyses	6
**Discussion**			
Key results	18	Summarise key results with reference to study objectives	7-11
Limitations	19	Discuss limitations of the study, taking into account sources of potential bias or imprecision. Discuss both direction and magnitude of any potential bias	12-13
Interpretation	20	Give a cautious overall interpretation of results considering objectives, limitations, multiplicity of analyses, results from similar studies, and other relevant evidence	13-14
Generalisability	21	Discuss the generalisability (external validity) of the study results	13-14
**Other information**			
Funding	22	Give the source of funding and the role of the funders for the present study and, if applicable, for the original study on which the present article is based	21

*Give information separately for cases and controls in case-control studies and, if applicable, for exposed and unexposed groups in cohort and cross-sectional studies.

Note: An Explanation and Elaboration article discusses each checklist item and gives methodological background and published examples of transparent reporting. The STROBE checklist is best used in conjunction with this article (freely available on the Web sites of PLoS Medicine at http://www.plosmedicine.org/, Annals of Internal Medicine at http://www.annals.org/, and Epidemiology at http://www.epidem.com/). Information on the STROBE Initiative is available at www.strobe-statement.org.

For peer review only

For peer review only - http://bmjopen.bmj.com/site/about/guidelines.xhtml
